# Recent Developments in C–H Activation for Materials Science in the Center for Selective C–H Activation

**DOI:** 10.3390/molecules23040922

**Published:** 2018-04-16

**Authors:** Junxiang Zhang, Lauren J. Kang, Timothy C. Parker, Simon B. Blakey, Christine K. Luscombe, Seth R. Marder

**Affiliations:** 1 School of Chemistry & Biochemistry, Georgia Institute of Technology, Atlanta, GA 30332, USA; jzhang84@mail.gatech.edu (J.Z.); timothy.parker@chemistry.gatech.edu (T.C.P.); 2 Department of Chemistry, University of Washington, Seattle, WA 98195, USA; KangL@uw.edu; 3 Department of Chemistry, Emory University, Atlanta, GA 30322, USA; 4 Department of Material Science and Engineering, University of Washington, Seattle, WA 98195, USA

**Keywords:** **Keywords**: π-conjugated materials, electron-acceptors, direct arylation, C–H activation, living polymerization

## Abstract

Organic electronics is a rapidly growing field driven in large part by the synthesis of π-conjugated molecules and polymers. Traditional aryl cross-coupling reactions such as the Stille and Suzuki have been used extensively in the synthesis of π-conjugated molecules and polymers, but the synthesis of intermediates necessary for traditional cross-couplings can include multiple steps with toxic and hazardous reagents. Direct arylation through C–H bond activation has the potential to reduce the number of steps and hazards while being more atom-economical. Within the Center for Selective C–H Functionalization (CCHF), we have been developing C–H activation methodology for the synthesis of π-conjugated materials of interest, including direct arylation of difficult-to-functionalize electron acceptor intermediates and living polymerization of π-conjugated polymers through C–H activation.

## 1. Introduction

Carbon–carbon cross-coupling reactions have proven indispensable for the construction of both small molecule and polymer organic semiconducting materials [1]. Advances in C–H functionalization catalysis now allow for the construction of certain materials directly from their precursor arenes [2,3,4]. However, in many cases, efficiencies and regioselectivities are not yet sufficiently high, so traditional cross-coupling strategies, in which the regioselectivity of the cross-coupling reaction is predetermined by the synthesis of appropriate organometallic nucleophiles and aryl halide electrophiles, remain important tools for the precise synthesis of novel materials. While many prefunctionalized building blocks are readily obtained by classical reactions (electrophilic aromatic substitution, aryl lithiation, etc.), an increased sophistication in the design of specialized materials, underpinned by a greater understanding of structure–activity relationships and computational predictions, demands a suite of more sophisticated building blocks. C–H functionalization reactions have emerged as a potential path to access novel building blocks that would be difficult to access using traditional chemistry. In addition, C–H functionalization reactions can provide more atom economic syntheses of important materials and building blocks, in many cases using fewer toxic or highly reactive reagents. 

In 2012, a materials effort as part of the Center for Selective C–H Functionalization (CCHF) funded by the United States National Science Foundation was tasked with developing C–H activation chemistry on novel or difficult-to-functionalize intermediates of interest to materials science as well as developing routes for potentially living or living-like C–H activated polymerization. This relatively short feature article mainly reviews the work related to materials chemistry within the NSF-CCHF. As this is a relatively focused review, and since application of C–H functionalization in materials chemistry is a fast-paced and emerging field, the authors regret that it is not possible for us to list all the significant contributions in this field and encourage interested readers to explore other excellent reviews on this topic [5,6,7].

## 2. Direct Arylation of Electron-Poor π-Conjugated Small Molecules

π-Conjugated small molecules are used for the fabrication of a variety of organic electronic devices [8] such as dye-sensitized solar cells (DSCs) [9], organic field-effect transistors (OFETs) [10], and organic solar cells (OSCs) [11,12] and are used extensively in liquid crystalline semiconductors [13] and non-linear optical materials [14]. Among the variety of π-conjugated systems reported, electron-donating (D) and electron-accepting units (A) are two key building blocks. By the judicious selection and assembly of varieties of D/A groups along the same backbone, one can make chromophores with desired absorption bandgaps, tailored electron affinity (EA) and ionization energy (IE), and favorable intra/inter-miscibility with surrounding materials.

Electron acceptor building blocks that have relatively high electron affinities and potentially strong intramolecular electron coupling with donor and/or other electron acceptor building blocks are necessary to be able to tune the properties of π-conjugated materials. Properties of specific interest include optical absorption and emission wavelengths, excited state oxidation, reduction potential (e.g., approximate EA), and oxygen stability in electron-transport materials. Practically, one can design electron-accepting units or molecules through two strategies. (1) Installment of π-withdrawing substituents such as cyano, carbonyls, dicyanovinyl, and the like, on the π-system will typically lower the energy of the lowest unoccupied molecular orbitals (LUMO) and have a smaller impact on the highest occupied molecular orbital (HOMO) levels. This is because the π* energy of the π-withdrawing substituent is often relatively close to that of the LUMO of the unsubstituted conjugated π-system, and the orbitals can thus mix efficiently, leading to stabilization of the LUMO of the electron acceptor building block. (2) Attachment of groups that are predominately inductively electron withdrawing, e.g.*,* perfluoroalkyl groups, can also make a system a stronger electron acceptor. Such substitution indirectly impacts the energy of the π-orbitals by effectively lowering the electron density at the nuclei of the atoms in the π-systems. Therefore, as a consequence, the π-orbitals become less effectively screened and both LUMO and HOMO will tend to be lowered. Similarly, substitution of an atom in a π-system with a more electron-negative atom, such as replacing a carbon of benzene with nitrogen as in pyridine (electronegativity of nitrogen is 3.07 versus 2.50 for carbon), will potentially lower HOMOs and LUMOs.

Manipulation of functional groups on π-electron acceptor units is one of the more challenging topics in traditional organic chemistry. Because of their intrinsically high electron affinity, electrophilic substitutions reactions such as halogenation are difficult to achieve. Additionally, deprotonation to form anions, e.g., to make Stille or Suzuki reagents, often results in nucleophilic addition to the π-electron acceptor and subsequent decomposition. Alternatively, transition metal catalyzed C–H bond functionalization may provide a path to functionalizing strong π-acceptor building blocks without the need for electrophilic substitution or strongly nucleophilic intermediates. Such metal-mediated reactions, often referred to as direct arylation, have been elegantly applied to electron donor building blocks such as thiophene for small molecule synthesis [3] and even the synthesis of high molecular weight polymers, which requires high starting monomer consumption, regioselectivity, and effectively no deleterious side-reactions [15]. Regarding π-electron acceptor substrates, seminal work by Fagnou and coworkers [16,17] on direct arylation of fluorinated arenes suggested that such reactions occur via a concerted metallation–deprotonation pathway (CMD) on the transition metal that avoids anionic aryl intermediates that are typically highly nucleophilic. These reactions in some cases may favor protons in more acidic substrates. This combination of non-nucleophilic intermediates and possible preference for more acidic substrates in certain cases suggested the possibility that such direct arylation might be useful in the functionalization of strong electron acceptors.

Direct C–H bond functionalization reactions are limited by two fundamental challenges: (i) reactivity, the inert nature of most carbon–hydrogen bonds, and (ii) selectivity—the requirement to control regioselectivity in molecules that contain diverse C–H groups. Two main advantages of strong electron acceptors that make them potential candidates for transition metal catalyzed C–H bond functionalization are (i) electronegative elements in electron-acceptor heteroarenes that render certain protons more acidic, which can be abstracted via CMD processes on the metal, and (ii) electron acceptors substituted with carbonyl-related groups (ester, amide, imide, and ketone), which may provide an effective directing group for C–H activation via cyclometallated intermediates. In the following sections, C–H activation of four types of relatively strong electron acceptor building blocks are presented along with their applications in organic electronics. 

### Benzothiadiazole Derivatives

**Scheme 1:**
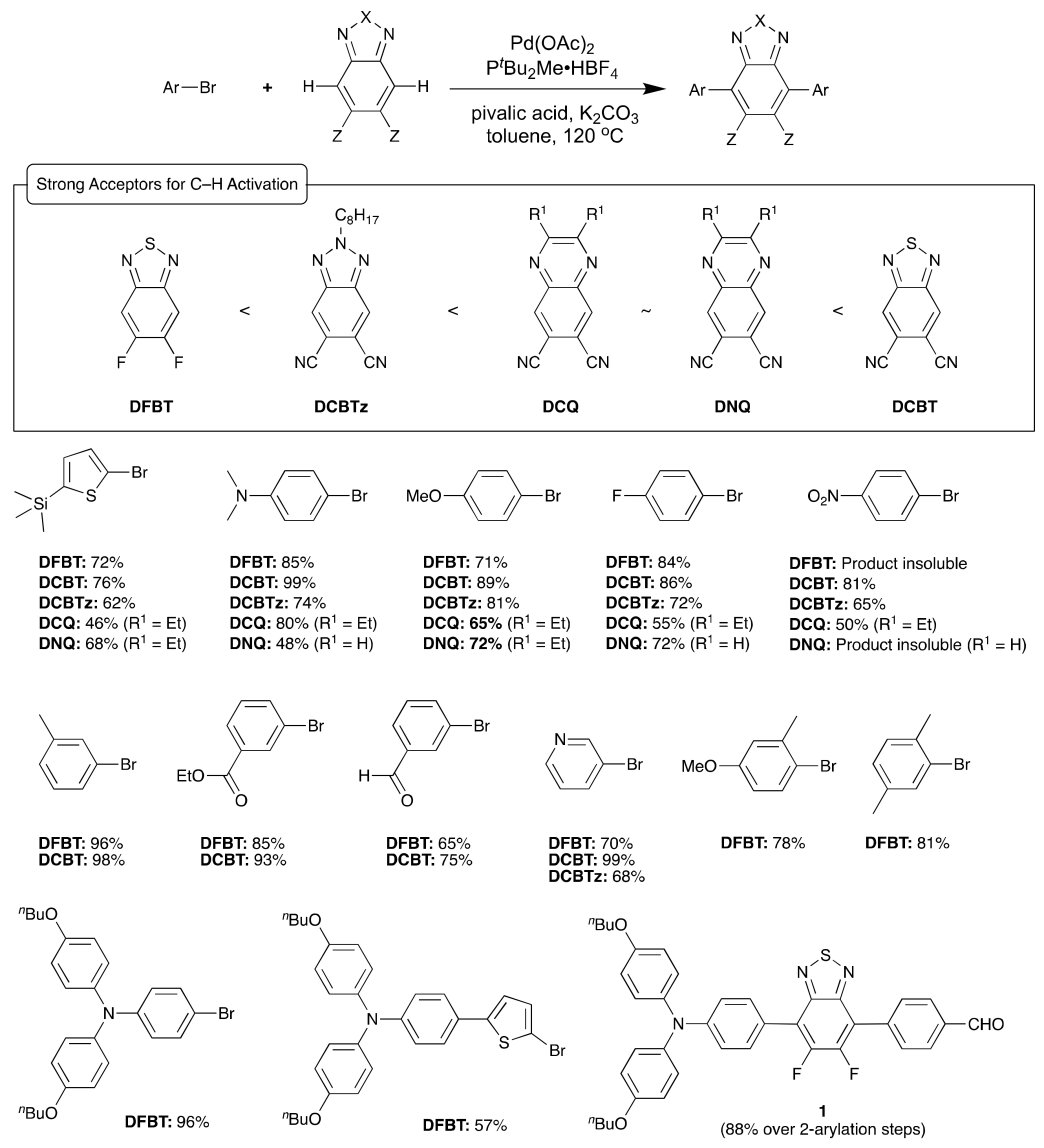
Strong electron acceptors for C–H activation with selected aryl halide substrates. Isolated reaction yields for disubstituted acceptor are given. The acceptors appear from left to right in order of increasing estimated electron affinity. In a few cases, the product of the coupling was insoluble in common organic solvents, which are noted.

Derivatives of 2,1,3-benzothiadiazole (BT), especially 5,6-difluorobenzothiadiazole (DFBT) derivatives, have been widely used in organic electronics [8] because of their relatively high electron affinity and planar structures that are favorable to intra- and intermolecular π-coupling. Given the reported harsh conditions for halogenating DFBT [18,19] or an alternative multistep synthesis [20], DFBT and potentially other acceptors with higher electron affinity than DFBT were chosen as targets for C–H activated direct arylation [21]. Scheme 1 summarizes a relatively effective coupling strategy that was developed based on the chemistry of Fagnou and coworkers [17] for DFBT and the four electron acceptors: 5,6-dicyanobenzo[*d*][1,2,3]triazole (DCBTz), 6,7-dicyanoquinoxaline (DCQ), 6,7-dinitroquinoxaline (DNQ), and 5,6-dicyano[2,1,3]benzothiadiazole (DCBT). The relative electron affinity of the electron acceptors was determined to be DFBT < DCBTz < DCQ~DNQ < DCBT based on electrochemical and optical measurements [21]. The method proved efficient for a variety of electron-donating and electron-accepting aryl halide substrates and good-to-excellent yields are generally obtained. It should be noted that, under the conditions shown in Scheme 1, C–H activation on the BT alone resulted in multiple arylation at the 5 and 6 positions in addition to the targeted 4 and 7 positions; however, Doucet and co-workers recently reported a phosphine-ligand free mono- and dual C–H activation of 2,1,3-benzothiadiazole in DMA solvent with high-to-moderate yields [22]. Unwanted over-arylation of the products was also seen when coupling the acceptors with 2-bromothiophenes having hydrogen in the 5-positions, which was addressed by the use of 2-bromo-5-(trimethylsilyl)thiophenes. Through proper selection of excess stoichiometry of the acceptor, monoarylated products could be obtained that then could be isolated and arylated again to yield differentially substituted materials with respect to the acceptor moiety such as **1** in Scheme 1. Differentially substituted BT and BTz derivatives have also been obtained by Zhang and coworkers via selective direct arylation of monobromo BT with aryl iodides or by direct arylation of using an ~3-fold excess of acceptor to the aryl iodide [23]. Zhang and coworkers also reported the direct olefination of BT derivatives with Pd(OAc)_2_ using silver(I) acetate and benzoquinone as co-oxidants to produce a variety of fluorescent compounds [24].

**Figure 1:**
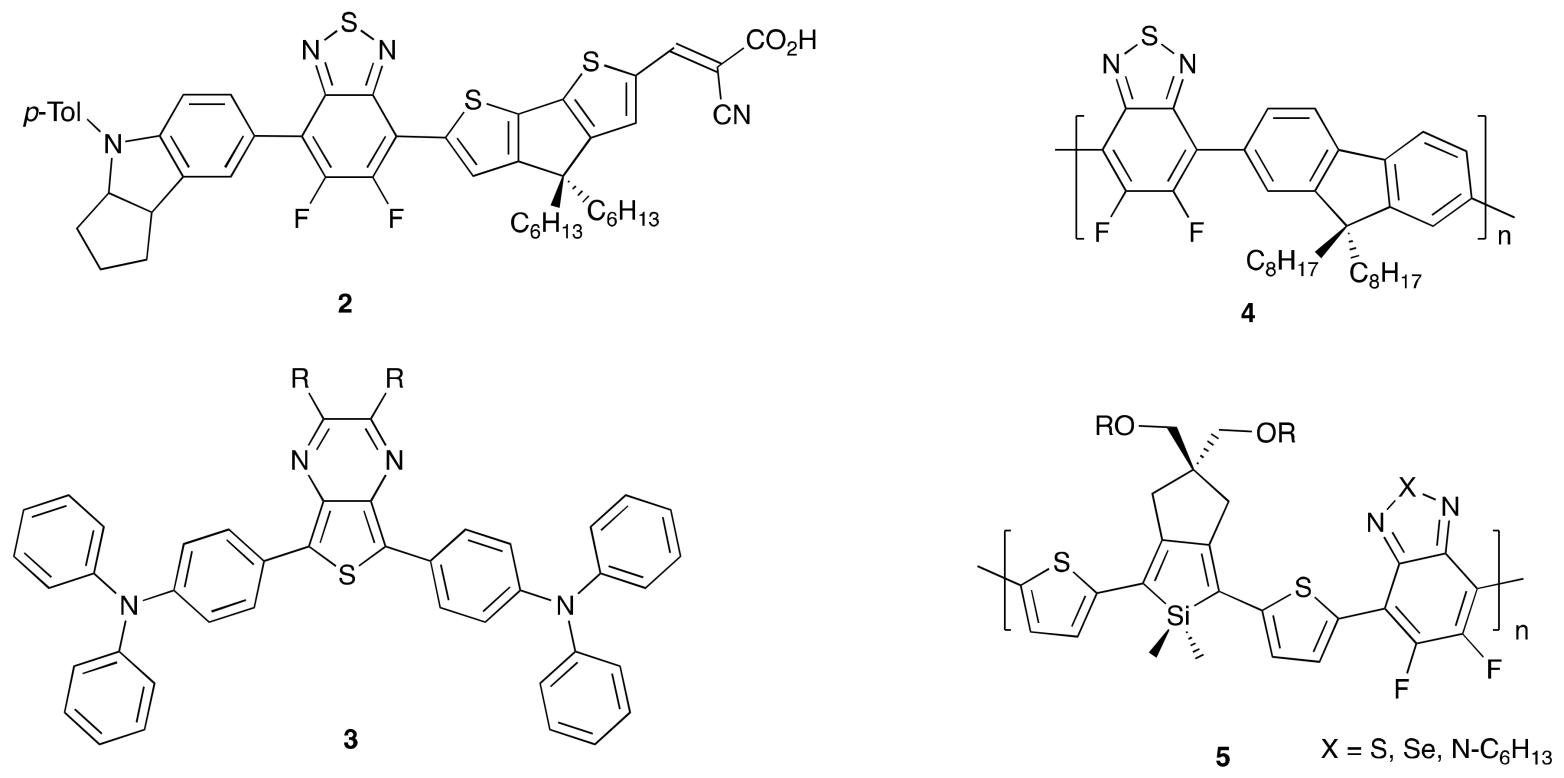
π-Conjugated materials synthesized by C–H activated direct arylation similar to that reported in Scheme 1.

The conditions summarized in Scheme 1 have been used in the synthesis of materials **2**–**5** shown in Figure 1. Sequential arylations of DFBT were utilized to make a series of D-A-π-A dyes such as **2** in Figure 1, incorporating both the donor triarylamine and the π-bridge cyclopentadithiophene (CPDT, as the monoaldehyde), which, after Knoevenagel condensation with cyanoacetic acid, gave chromophores that were incorporated into DSSCs with efficiency up to ~9% [25,26]. Similar conditions have also been used for C–H activation of thienopyrazines such as **3** (Figure 1) to yield dicoupled D-A-D chromophores for NIR emitting materials in moderate to high yields [27]. Zhang and coworkers thoroughly optimized C–H coupling conditions between DFBT and dioctylfluorene monomers (21 reaction variations reported) to find similar coupling conditions as shown in Scheme 1 for direct arylation polymerization (DArP) to yield **4** (Figure 1) [28]. Notably, polymerization seemed to stop via reductive debromination and use of BT or 5,6-dialkoxy BT monomers in the polymerization resulted in no significant reaction, suggesting a preference for the electron-accepting substrate DFBT. Under optimized DArP conditions, DFBT Polymer **4** was obtained with an *M_n_* ~41 kg/mol in an 86% yield. Strong electron acceptors were also incorporated into silole-containing polymers such as **5** (Figure 1) via C–H activation by Scott and coworkers with a maximum *M_n_* of ~13 kg/mol for coupling with 2,5-di-(4′-bromothienyl)silole derivatives using modified DArP conditions (Pd(dba)_3_•CHCl_3_/tris(*o*-methoxyphenyl)phosphine) and with a yield of up to 80% [29].

## 3. Earth-Abundant Metal-Initiated Iodination

**Scheme 2:**
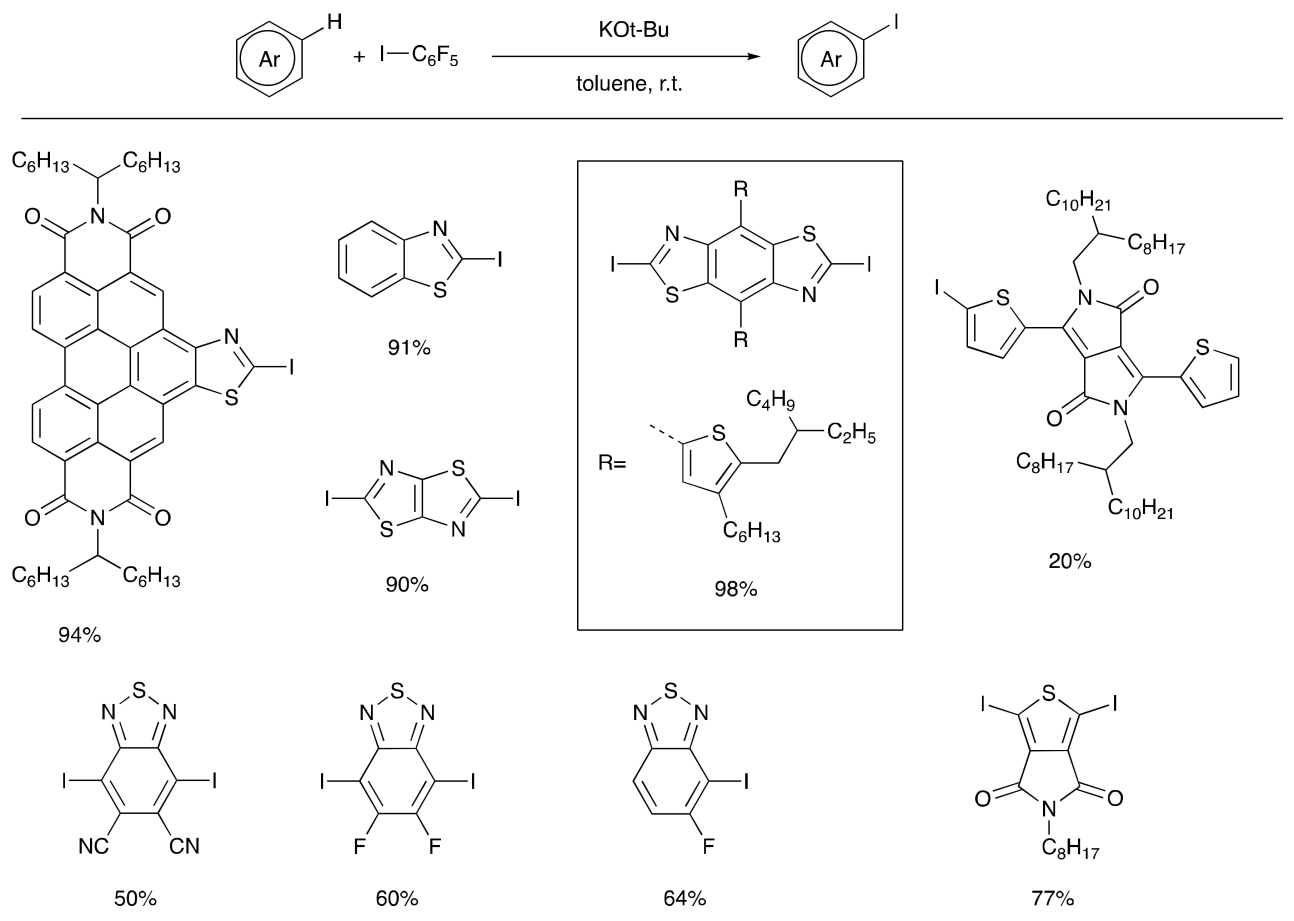
Potassium *tert*-butoxide initiated C–H iodination of electron-deficient heteroaromatic compounds.

Other advances in C–H functionalization now allow for the construction of certain intermediates useful in traditional cross coupling reactions directly from the precursor arenes such as Suzuki reagents [30] and aryl silanes [31] via relatively mild catalytic pathways. Such silanes may be used directly in Hiyama coupling [32] or Denmark-Hiyama coupling [33], or in turn may be converted to aryl halides, Suzuki reagents, Stille reagents, etc. In a recent advance, potassium *tert*-butoxide was shown to promote efficient C–H silylation of heteroaromatic systems [34,35,36]. A similar potassium *tert*-butoxide radial initiation concept has also been applied to the C–H iodination of electron acceptor arenes (Scheme 2) [37], which are typically resistant to electrophilic aromatic substitution and prone to reduction by organometallic reagents such as alkyl lithium and Grignard reagents that might be employed to deprotonate them. The new C–H iodination reaction proceeds at room temperature, and in many cases a sub-stoichiometric quantity of potassium *tert*-butoxide is sufficient to provide good reaction yields. In cases where low yields are observed under standard reaction conditions, the use of excess potassium *tert*-butoxide and pentafluorophenyliodide often lead to significant improvements. Highlights noted in Scheme 2 include the iodination of a range of thiazole derivatives, including a particularly electron-deficient thiazoloperylenediimide, and several benzothiadiazole derivatives. In the case of the mono-fluorobenzothiadiazole, excellent regioselectivity in the iodination reaction is observed to yield the single regioisomer shown.

**Figure 2:**
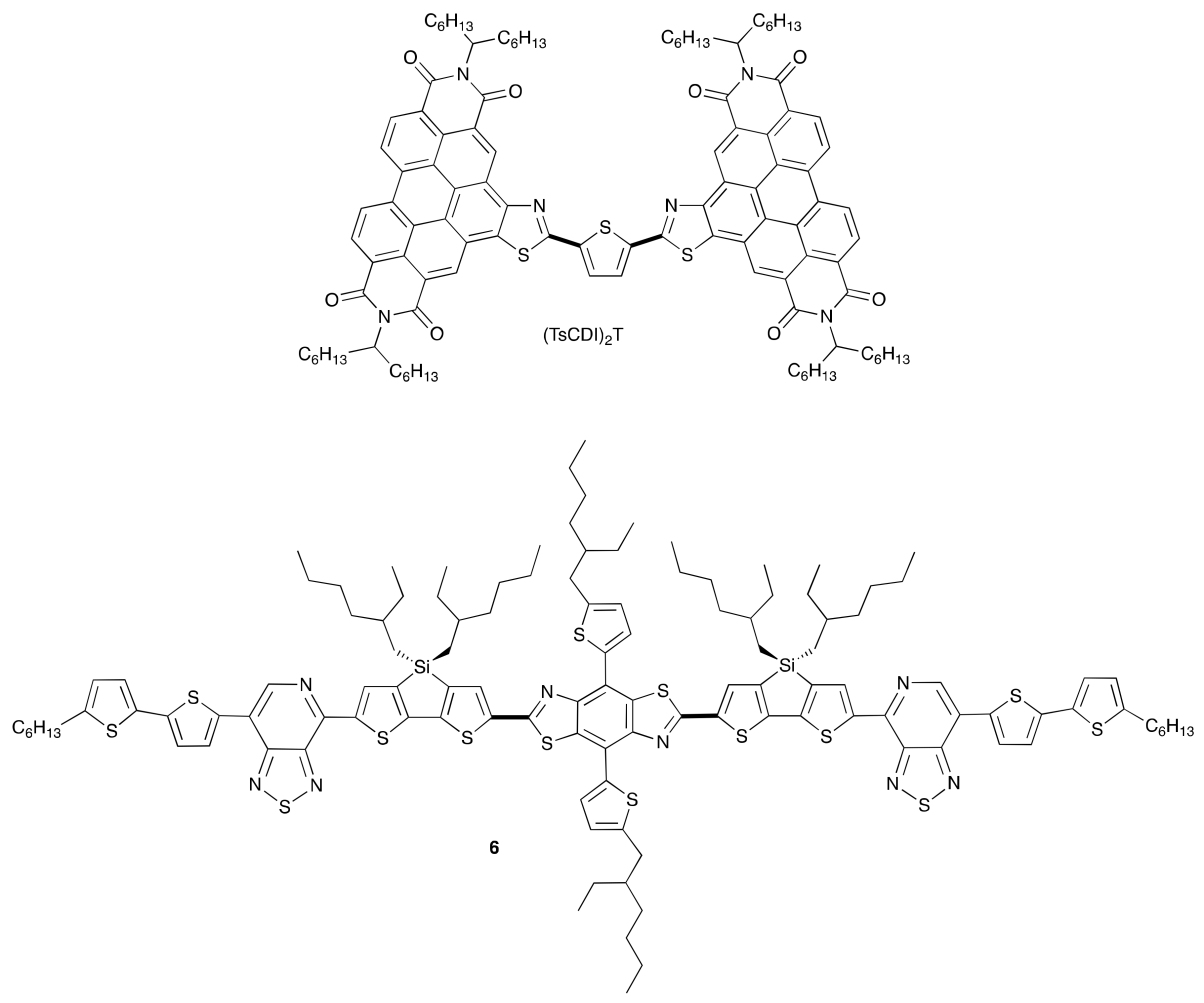
Materials constructed using C–H iodination methodology for application in organic photovoltaics. Bold lines indicate bonds formed by C–H activated direct arylation.

Subsequent to this discovery of the *tert*-butoxide initiated C–H iodination, additional variations on the reaction conditions, including different initiators [38], and different halogen sources have been developed. The C–H iodination reaction has been utilized in the synthesis of the non-fullerene acceptor (TsCDI)_2_T (Figure 2), which demonstrated promising properties as an electron transport material in an organic bulk heterojunction (BHJ) solar cell [37,39]. Subsequently, a direct C–H arylation of the parent PDI-thiazole has been developed enabling a modular synthesis of a number of (TsCDI)_2_ analogues [39]. Additionally, the application of this reaction in the synthesis of intermediate-sized conjugated donor molecules such as **6** (Figure 2), also for application in BHJ solar cells, has been reported [40]. 

## 4. Toward Living Polymerizations to Synthesize Semiconducting Polymers Using Poly(3-hexylthiophene) as the Model Polymer

Poly(3-hexylthiophene) (P3HT) is one of the most widely used and studied organic electronic polymers partly due to the facile synthesis of specific molecular weight polymers with narrow dispersity (*Đ*). These properties traditionally result from the use of organometallic monomers, synthesized from their respective aryl halide precursors. In 2011, Mori et al. presented a shortcut to access the organometallic thiophene precursor of P3HT via deprotonation of the thiophene, in which an alkyl magnesium halide and a catalytic amount of an amine base were reacted with 2-chloro-3-hexyl-thiophene to generate the Grignard-monomer prior to polymerization. While this reaction was not enabled by CH-activation, it eliminated the need for a secondary halogenation of the thiophene monomer and used a catalytic amount of base, highlighting the utility to develop more atom-efficient methods to synthesize P3HT [41]. In addition, direct arylation polymerization (DArP) has grown popular for the synthesis of conjugated polymers, but these reactions often lack control over molecular weight and dispersity [42,43,44]. Therefore, our aim was to design a controlled DArP method with minimal number of steps prior to the actual polymerization, while removing reactive or sensitive reagents such as Grignard compounds. We were attracted to the idea of dual-metal catalysis, where two different metals with orthogonal reactivity are used to selectively activate different steps of the polymerization process [45,46]. Homogeneous gold catalysis has been widely investigated in recent years [47,48,49]. The attractive features of gold(I) halide compounds are their unlikeliness to undergo oxidative addition with carbon–halogen bonds of aromatic compounds [50,51,52,53], and their ability to insert into electron-deficient aromatic and heteroaromatic C−H bonds [54,55,56,57], including the C−H activation of bromothiophenes under basic conditions [58]. An additional advantage is the ability of gold(I) compounds to undergo transmetalation with palladium(II) complexes [59,60,61,62,63], which are commonly utilized to catalyze the C–C coupling of thiophene monomers. 

**Scheme 3:**
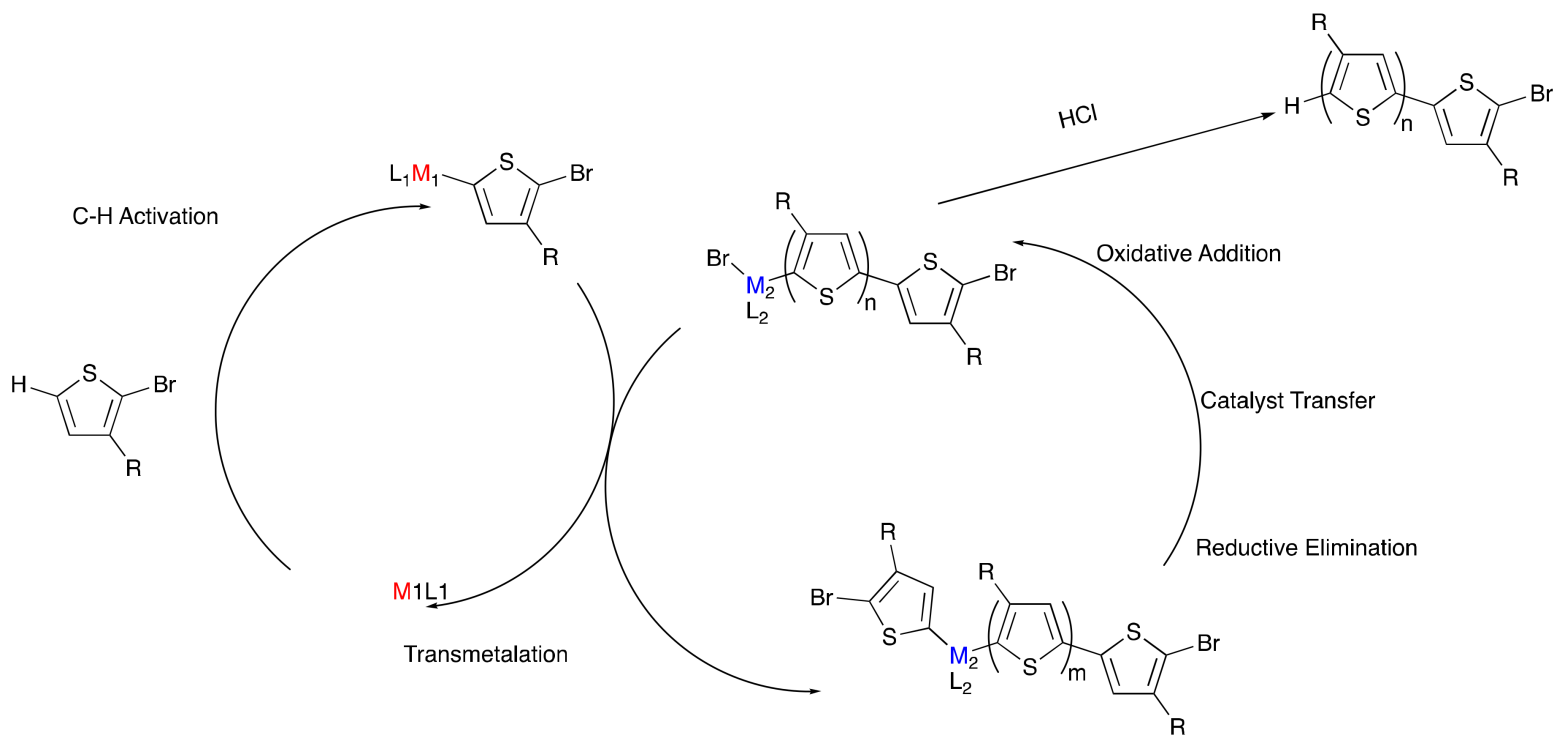
Proposed dual-metal catalyzed poly(3-hexylthiophene) (P3HT) synthesis.

**Scheme 4:**
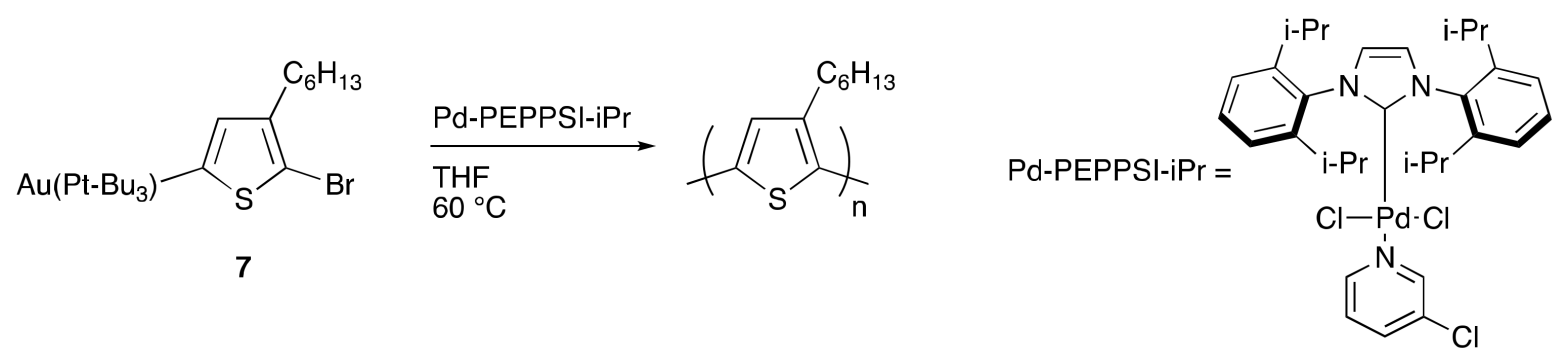
Polymerization of aurylated thiophene species.

To model the concept of dual-metal catalyzed P3HT synthesis (Scheme 3), a gold and palladium system was explored [64]. First chloro(tri-tert-butylphosphine)gold(I) was utilized to C–H activate and aurylate the 5-position of 2-bromo-3-hexylthiophene, resulting in Monomer **7** (Scheme 4). Monomer **7** was then purified and isolated, demonstrating the remarkable stability of the product, especially in comparison to its traditional Grignard counterpart. The purified monomer was polymerized to P3HT using Pd-PEPPSI-iPr as a catalyst (Scheme 4). The polymerization indicated successful transmetalation and cross-coupling of the aurylated thiophene monomer by the palladium catalyst. In addition, the resulting polymers closely matched the theoretical molecular weights based on the monomer-to-catalyst ratio while also exhibiting narrow dispersities, which correlates with controlled chain-growth polymerization. Upon further investigation, a linear relationship was observed between molecular weight and monomer conversion indicating a single catalyst association to a single polymer chain. The narrow dispersities observed also support a controlled polymerization. The addition of a second equivalent of monomer, after the consumption of the initial monomer, resulted in a predicted doubling of the molecular weight, further supporting the living nature of the polymerization. The good accessibility of **7** via C–H activation and its ability to undergo controlled palladium-catalyzed polymerization open new avenues to explore dual-catalytic DArP with two metals of orthogonal reactivity, without the use of often sensitive organometallic chemicals.

**Scheme 5:**
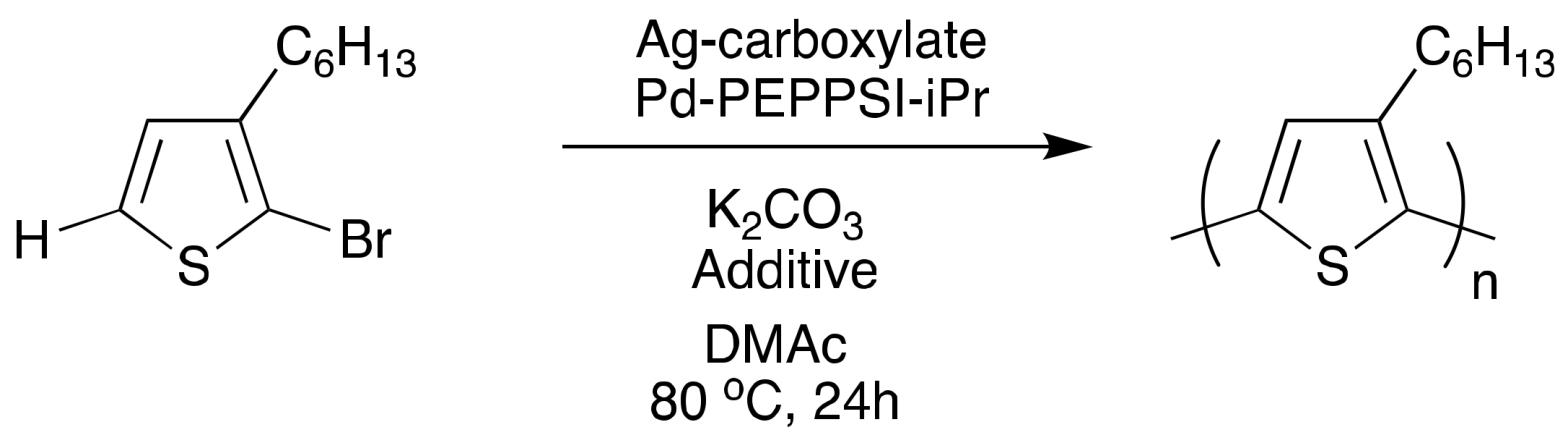
One-pot dual-catalytic silver and palladium polymerization.

Moving forward, a dual-catalytic, one-pot polymerization was attempted using the reported gold-palladium conditions; however, polymerization was unfeasible due to hypothesized incompatibilities of the reagents needed for each cycle (Scheme 3). Silver was explored as another catalyst option, due to promising evidence for its ability to promote C–H activation [65] and to transmetalate with palladium. One-pot reactions (Scheme 5) resulted in successful polymer synthesis. In the absence of any silver species, polymerization does occur, in which palladium catalyzes both the C–H activation and the C–C coupling. The resulting polymer, however, has low molecular weight and broad dispersity. Incorporation of silver(I) adamantanoate (AgAd) resulted in increased molecular weight, while the use of silver(I) neodecanoate (AgNDA) resulted in improved regioregularity. Reactions with AgAd provided polymers with narrower dispersities compared to reactions using AgNDA, which resulted in bimodal distributions. Adding 1 equivalent of pyridine with respect to the monomer narrowed the dispersity (*Đ*~1.3–1.4) and yielded unimodality (in the AgNDA system). This was indicative of pyridine’s suppression of the number of active species of PEPPSI-iPr, allowing for C–H activation to be primarily silver-mediated, promoting orthogonal reactivity of the two metals, thus yielding chain-growth kinetics. By incorporating both silver for C–H activation and palladium for transmetalation and C–C coupling, chain-growth kinetics with low dispersities (<1.5) were realized, which are not typically observed characteristics of DArP P3HT syntheses [66,67,68]. 

This is the first reported dual-catalytic silver-palladium system for P3HT synthesis via DArP [69]. The observation of chain-growth, low dispersities, lack of chain transfer, and regioregularity values up to 96% are significant steps toward pursuing a controlled polymerization via DArP. PEPPSI-iPr and silver-carboxylates also possess high chemical stability as opposed to alternative organometallics, which will facilitate less stringent reaction conditions that could have beneficial impacts on ease of synthesis and synthetic scalability. To further this pursuit, understanding the role of active catalytic species and their reaction kinetics could help explain the current lack of control over the molecular weight. Computational mechanistic insight could also aid in the screening of new ligand, additive, and catalyst moieties.

## 5. Conclusions and Outlook

Although over the last decade a substantial amount of progress has been made by many research groups around the world on the application of C–H activation in materials chemistry, a number of challenges remain and others maybe should be considered. Among those are (1), in aryl halide/thiophene C–H coupling, a higher selectivity for the desired aryl-thiophene bond and less thiophene-thiophene coupling and in particular less coupling of the thiophene in the β-positions, which ideally should effectively be eliminated in favor of high α-thiophene coupling, (2) oxidative C–H/C–H homocoupling, and in particular cross coupling, of both electron acceptors and electron donors in any combination with high selectivity such that excess stoichiometry of one coupling partner is unnecessary and in all cases where oxygen can be used as the terminal oxidant (instead of often large excess of silver(I) salts), (3) post-synthesis diversification of molecules and polymers using other C–H functionalization chemistry that is highly selective for specific sites either on alkyl pendant groups or the aryl core/backbones that would allow probing and optimization of side-chain engineering on morphology and nanoscale intermolecular structure without the need to synthesize a daunting array of intermediates and monomers, (4) use of first row and earth-abundant transition metal catalysts in place of commonly used Pd catalysts, (5) performing the reactions in water, and (6) metal-free synthesis of organic electronic materials. Meeting these challenges, other challenges, and challenges yet to be identified or discovered not only will be critical in providing more sustainable materials development but may also allow researchers to conceive of new materials and research plans with increased breadth that can help define and answer important scientific questions in organic electronic materials.

## References

[1] Marzano G., Ciasca C.V., Babudri F., Bianchi G., Pellegrino A., Po R., Farinola G.M. (2014). Organometallic Approaches to Conjugated Polymers for Plastic Solar Cells: From Laboratory Synthesis to Industrial Production. Eur. J. Org. Chem..

[2] Alberico D., Scott M.E., Lautens M. (2007). Aryl-aryl bond formation by transition-metal-catalyzed direct arylation. Chem. Rev..

[3] Schipper D.J., Fagnou K. (2011). Direct Arylation as a Synthetic Tool for the Synthesis of Thiophene-Based Organic Electronic Materials. Chem. Mater..

[4] Kowalski S., Allard S., Zilberberg K., Riedl T., Scherf U. (2013). Direct arylation polycondensation as simplified alternative for the synthesis of conjugated (co)polymers. Prog. Polym. Sci..

[5] Bohra H., Wang M. (2017). Direct C–H arylation: A “Greener” approach towards facile synthesis of organic semiconducting molecules and polymers. J. Mater. Chem. A.

[6] Pouliot J.R., Grenier F., Blaskovits J.T., Beaupre S., Leclerc M. (2016). Direct (Hetero)arylation Polymerization: Simplicity for Conjugated Polymer Synthesis. Chem. Rev..

[7] Suraru S.-L., Lee J.A., Luscombe C.K. (2016). C–H Arylation in the Synthesis of π-Conjugated Polymers. ACS Macro Lett..

[8] Guo X., Baumgarten M., Müllen K. (2013). Designing π-conjugated polymers for organic electronics. Prog. Polym. Sci..

[9] Hagfeldt A., Boschloo G., Sun L., Kloo L., Pettersson H. (2010). Dye-sensitized solar cells. Chem. Rev..

[10] Klauk H. (2010). Organic thin-film transistors. Chem. Soc. Rev..

[11] Zhou H., Yang L., You W. (2012). Rational Design of High Performance Conjugated Polymers for Organic Solar Cells. Macromolecules.

[12] Lin Y., Li Y., Zhan X. (2012). Small molecule semiconductors for high-efficiency organic photovoltaics. Chem. Soc. Rev..

[13] Iino H., Hanna J.-I. (2016). Liquid crystalline organic semiconductors for organic transistor applications. Polym. J..

[14] Dalton L.R., Sullivan P.A., Bale D.H. (2010). Electric field poled organic electro-optic materials: State of the art and future prospects. Chem. Rev..

[15] Mercier L.G., Leclerc M. (2013). Direct (hetero)arylation: A new tool for polymer chemists. Acc. Chem. Res..

[16] Lapointe D., Fagnou K. (2010). Overview of the Mechanistic Work on the Concerted Metallation–Deprotonation Pathway. Chem. Lett..

[17] Lafrance M., Rowley C.N., Woo T.K., Fagnou K. (2006). Catalytic intermolecular direct arylation of perfluorobenzenes. J. Am. Chem. Soc..

[18] Dou L., Chen C.-C., Yoshimura K., Ohya K., Chang W.-H., Gao J., Liu Y., Richard E., Yang Y. (2013). Synthesis of 5*H*-Dithieno[3,2-b:2′,3′-d]pyran as an Electron-Rich Building Block for Donor–Acceptor Type Low-Bandgap Polymers. Macromolecules.

[19] You W. (2011). Polymers with Tunable Band Gaps for Photonic and Electronic Applications. WO Patent.

[20] Zhang Y., Chien S.C., Chen K.S., Yip H.L., Sun Y., Davies J.A., Chen F.C., Jen A.K.-Y. (2011). Increased open circuit voltage in fluorinated benzothiadiazole-based alternating conjugated polymers. Chem. Commun..

[21] Zhang J., Parker T.C., Chen W., Williams L., Khrustalev V.N., Jucov E.V., Barlow S., Timofeeva T.V., Marder S.R. (2016). C–H-Activated Direct Arylation of Strong Benzothiadiazole and Quinoxaline-Based Electron Acceptors. J. Org. Chem..

[22] Idris I., Tannoux T., Derridj F., Dorcet V., Boixel J., Guerchais V., Soulé J.-F., Doucet H. (2018). Effective modulation of the photoluminescence properties of 2,1,3-benzothiadiazoles and 2,1,3-benzoselenadiazoles by Pd-catalyzed C–H bond arylations. J. Mater. Chem. C.

[23] He C.Y., Wu C.Z., Qing F.L., Zhang X. (2014). Direct (het)arylation of fluorinated benzothiadiazoles and benzotriazole with (het)aryl iodides. J. Org. Chem..

[24] Xiao Y.L., Zhang B., He C.Y., Zhang X. (2014). Direct olefination of fluorinated benzothiadiazoles: A new entry to optoelectronic materials. Chem. Eur. J..

[25] Kang X., Zhang J., O’Neil D., Rojas A.J., Chen W., Szymanski P., Marder S.R., El-Sayed M.A. (2014). Effect of Molecular Structure Perturbations on the Performance of the D-A-π-A Dye Sensitized Solar Cells. Chem. Mater..

[26] Kang X., Zhang J., Rojas A.J., O′Neil D., Szymanski P., Marder S.R., El-Sayed M.A. (2014). Deposition of loosely bound organic D-A-π-A′ dyes on sensitized TiO_2_ film: A possible strategy to suppress charge recombination and enhance power conversion efficiency in dye-sensitized solar cells. J. Mater. Chem. A.

[27] McNamara L.E., Liyanage N., Peddapuram A., Murphy J.S., Delcamp J.H., Hammer N.I. (2016). Donor-Acceptor-Donor Thienopyrazines via Pd-Catalyzed C–H Activation as NIR Fluorescent Materials. J. Org. Chem..

[28] Zhang X., Gao Y., Li S., Shi X., Geng Y., Wang F. (2014). Synthesis of poly(5,6-difluoro-2,1,3-benzothiadiazole-alt-9,9-dioctyl-fluorene) via direct arylation polycondensation. J. Polym. Sci. Part A Polym. Chem..

[29] Scott C.N., Bisen M.D., Stemer D.M., McKinnon S., Luscombe C.K. (2017). Direct Arylation Polycondensation of 2,5-Dithienylsilole with a Series of Difluorobenzodiimine-Based Electron Acceptors. Macromolecules.

[30] Mkhalid I.A., Barnard J.H., Marder T.B., Murphy J.M., Hartwig J.F. (2010). C–H activation for the construction of C-B bonds. Chem. Rev..

[31] Cheng C., Hartwig J.F. (2015). Catalytic Silylation of Unactivated C–H Bonds. Chem. Rev..

[32] Hatanaka Y., Hiyama T. (1988). Cross-coupling of organosilanes with organic halides mediated by a palladium catalyst and tris(diethylamino)sulfonium difluorotrimethylsilicate. J. Org. Chem..

[33] Denmark S.E., Regens C.S. (2008). Palladium-catalyzed cross-coupling reactions of organosilanols and their salts: Practical alternatives to boron- and tin-based methods. Acc. Chem. Res..

[34] Toutov A.A., Liu W.B., Betz K.N., Fedorov A., Stoltz B.M., Grubbs R.H. (2015). Silylation of C–H bonds in aromatic heterocycles by an Earth-abundant metal catalyst. Nature.

[35] Liu W.B., Schuman D.P., Yang Y.F., Toutov A.A., Liang Y., Klare H.F.T., Nesnas N., Oestreich M., Blackmond D.G., Virgil S.C. (2017). Potassium *tert*-Butoxide-Catalyzed Dehydrogenative C–H Silylation of Heteroaromatics: A Combined Experimental and Computational Mechanistic Study. J. Am. Chem. Soc..

[36] Banerjee S., Yang Y.F., Jenkins I.D., Liang Y., Toutov A.A., Liu W.B., Schuman D.P., Grubbs R.H., Stoltz B.M., Krenske E.H. (2017). Ionic and Neutral Mechanisms for C–H Bond Silylation of Aromatic Heterocycles Catalyzed by Potassium *tert*-Butoxide. J. Am. Chem. Soc..

[37] Shi Q.Q., Zhang S.Y., Zhang J.X., Oswald V.F., Amassian A., Marder S.R., Blakey S.B. (2016). (KOBu)-Bu-*t*-Initiated Aryl C–H Iodination: A Powerful Tool for the Synthesis of High Electron Affinity Compounds. J. Am. Chem. Soc..

[38] Liu X., Zhao X., Liang F., Ren B. (2018). *t*-BuONa-mediated direct C–H halogenation of electron-deficient (hetero)arenes. Org. Biomol. Chem..

[39] Shi Q.Q., Andreansky E.S., Marder S.R., Blakey S.B. (2017). Synthesis and C–H Functionalization Chemistry of Thiazole-Semicoronenediimide (TsCDIs) and -Coronenediimides (TCDIs). J. Org. Chem..

[40] Zhang S.Y., Zhang J.X., Abdelsamie M., Shi Q.Q., Zhang Y.D., Parker T.C., Jucov E.V., Timofeeva T.V., Amassian A., Bazan G.C. (2017). Intermediate-Sized Conjugated Donor Molecules for Organic Solar Cells: Comparison of Benzodithiophene and Benzobisthiazole-Based Cores. Chem. Mater..

[41] Tamba S., Shono K., Sugie A., Mori A. (2011). C–H functionalization polycondensation of chlorothiophenes in the presence of nickel catalyst with stoichiometric or catalytically generated magnesium amide. J. Am. Chem. Soc..

[42] Facchetti A., Vaccaro L., Marrocchi A. (2012). Semiconducting polymers prepared by direct arylation polycondensation. Angew. Chem. Int. Ed..

[43] Okamoto K., Zhang J., Housekeeper J.B., Marder S.R., Luscombe C.K. (2013). C–H Arylation Reaction: Atom Efficient and Greener Syntheses of π-Conjugated Small Molecules and Macromolecules for Organic Electronic Materials. Macromolecules.

[44] Segawa Y., Maekawa T., Itami K. (2015). Synthesis of extended π-systems through C–H activation. Angew. Chem. Int. Ed..

[45] Perez-Temprano M.H., Casares J.A., Espinet P. (2012). Bimetallic catalysis using transition and Group 11 metals: An emerging tool for C-C coupling and other reactions. Chem. Eur. J..

[46] Lohr T.L., Marks T.J. (2015). Orthogonal tandem catalysis. Nat. Chem..

[47] Hashmi A.S., Hutchings G.J. (2006). Gold catalysis. Angew. Chem. Int. Ed..

[48] Furstner A., Davies P.W. (2007). Catalytic carbophilic activation: Catalysis by platinum and gold π acids. Angew. Chem. Int. Ed..

[49] Wegner H.A., Auzias M. (2011). Gold for C-C coupling reactions: A Swiss-Army-knife catalyst?. Angew. Chem. Int. Ed..

[50] Lauterbach T., Livendahl M., Rosellon A., Espinet P., Echavarren A.M. (2010). Unlikeliness of Pd-free gold(I)-catalyzed Sonogashira coupling reactions. Org. Lett..

[51] Livendahl M., Goehry C., Maseras F., Echavarren A.M. (2014). Rationale for the sluggish oxidative addition of aryl halides to Au(I). Chem. Commun..

[52] Joost M., Zeineddine A., Estevez L., Mallet-Ladeira S., Miqueu K., Amgoune A., Bourissou D. (2014). Facile oxidative addition of aryl iodides to gold(I) by ligand design: Bending turns on reactivity. J. Am. Chem. Soc..

[53] Guenther J., Mallet-Ladeira S., Estevez L., Miqueu K., Amgoune A., Bourissou D. (2014). Activation of aryl halides at gold(I): Practical synthesis of (P,C) cyclometalated gold(III) complexes. J. Am. Chem. Soc..

[54] Cambeiro X.C., Boorman T.C., Lu P., Larrosa I. (2013). Redox-controlled selectivity of C–H activation in the oxidative cross-coupling of arenes. Angew. Chem. Int. Ed..

[55] Boorman T.C., Larrosa I. (2011). Gold-mediated C–H bond functionalisation. Chem. Soc. Rev..

[56] Boogaerts I.I.F., Nolan S.P. (2010). Carboxylation of C–H bonds using N-heterocyclic carbene gold(I) complexes. J. Am. Chem. Soc..

[57] Gaillard S., Cazin C.S., Nolan S.P. (2012). N-heterocyclic carbene gold(I) and copper(I) complexes in C–H bond activation. Acc. Chem. Res..

[58] Ahlsten N., Perry G.J.P., Cambeiro X.C., Boorman T.C., Larrosa I. (2013). A silver-free system for the direct C–H auration of arenes and heteroarenes from gold chloride complexes. Catal. Sci. Technol..

[59] Hansmann M.M., Pernpointner M., Dopp R., Hashmi A.S. (2013). A theoretical DFT-based and experimental study of the transmetalation step in Au/Pd-mediated cross-coupling reactions. Chem. Eur. J..

[60] Hashmi A.S.K., Döpp R., Lothschütz C., Rudolph M., Riedel D., Rominger F. (2010). Scope and Limitations of Palladium-Catalyzed Cross-Coupling Reactions with Organogold Compounds. Adv. Synth. Catal..

[61] Del Pozo J., Casares J.A., Espinet P. (2013). The decisive role of ligand metathesis in Au/Pd bimetallic catalysis. Chem. Commun..

[62] Shi Y., Roth K.E., Ramgren S.D., Blum S.A. (2009). Catalyzed catalysis using carbophilic Lewis acidic gold and Lewis basic palladium: Synthesis of substituted butenolides and isocoumarins. J. Am. Chem. Soc..

[63] Perez-Temprano M.H., Casares J.A., de Lera A.R., Alvarez R., Espinet P. (2012). Strong metallophilic interactions in the palladium arylation by gold aryls. Angew. Chem. Int. Ed..

[64] Suraru S.-L., Lee J.A., Luscombe C.K. (2016). Preparation of an Aurylated Alkylthiophene Monomer via C–H Activation for Use in Pd-PEPPSI-iPr Catalyzed-Controlled Chain Growth Polymerization. ACS Macro Lett..

[65] Lotz M.D., Camasso N.M., Canty A.J., Sanford M.S. (2016). Role of Silver Salts in Palladium-Catalyzed Arene and Heteroarene C–H Functionalization Reactions. Organometallics.

[66] Wang Q., Takita R., Kikuzaki Y., Ozawa F. (2010). Palladium-catalyzed dehydrohalogenative polycondensation of 2-bromo-3-hexylthiophene: An efficient approach to head-to-tail poly(3-hexylthiophene). J. Am. Chem. Soc..

[67] Lai Y.-Y., Tung T.-C., Liang W.-W., Cheng Y.-J. (2015). Synthesis of Poly(3-hexylthiophene), Poly(3-hexylselenophene), and Poly(3-hexylselenophene-alt-3-hexylthiophene) by Direct C–H Arylation Polymerization via N-Heterocyclic Carbene Palladium Catalysts. Macromolecules.

[68] Rudenko A.E., Thompson B.C. (2015). Influence of the Carboxylic Acid Additive Structure on the Properties of Poly(3-hexylthiophene) Prepared via Direct Arylation Polymerization (DArP). Macromolecules.

[69] Lee J.A., Luscombe C.K. Dual-catalytic Ag-Pd System for a Chain-Growth Direct Arylation Polymerization to Synthesize Poly(3-hexylthiophene). ACS Macro Lett..

